# Neuroprotective effect of *Costus afer* on low dose heavy metal mixture (lead, cadmium and mercury) induced neurotoxicity via antioxidant, anti-inflammatory activities

**DOI:** 10.1016/j.toxrep.2020.08.008

**Published:** 2020-08-15

**Authors:** Brilliance O. Anyanwu, Chinna N. Orish, Anthonet N. Ezejiofor, Ify L. Nwaogazie, Orish E. Orisakwe

**Affiliations:** a African Centre of Excellence for Oilfield Chemicals Research (ACE-CEFOR), University of Port Harcourt, PMB, 5323 Port Harcourt, Choba, Nigeria; b Department of Anatomy, Faculty of Basic Medical Sciences, College of Health Sciences, University of Port Harcourt, PMB, 5323 Port Harcourt, Choba, Nigeria; c African Centre of Excellence for Public Health and Toxicological Research (ACE-PUTOR), University of Port Harcourt, PMB, 5323 Port Harcourt, Choba, Nigeria

**Keywords:** Neurotoxicity, Heavy metal exposure, Metal chelation, Oxidative stress, *Costus afer*

## Abstract

Humans are constantly exposed to heavy metals due to their ubiquity in the environment. Hence, this study investigated the possible protective effect of *Costus afer* aqueous leaf extract (CALE) against low dose heavy metal mixture (LDHMM)-induced neurotoxicity. Male albino rats were divided into 6 equal groups. Group 1 served as the normal control receiving only deionized water. Group 2 served as the toxic control receiving on metal mixture (20 mg/kg PbCl_2_, 1.61 mg/kg CdCl_2_ and 0.40 mg/kg HgCl_2_), groups 3, 4 and 5 were co-treated with metal mixture and CALE (750, 1500 and 2250 mg/kg body weight, respectively) and group 6 was treated with metal mixture and ZnCl_2_. All treatments were administered through oral gavage for 90days. Oxidative stress biomarkers [malondialdehyde (MDA), superoxide dismutase (SOD), glutathione content (GSH) and catalase (CAT)], inflammatory cytokines [interlukin-6 (IL-6) and interlukin-10 (IL-10)], histopathological changes and heavy metal concentration were determined in brain of rats. Results indicated that LDHMM significantly increased (p < 0.05) the lipid peroxidation marker (MDA) and the pro-inflammatory cytokine (IL-6), while lowered levels of the oxidative biomarkers (SOD, CAT and GSH) and anti-inflammatory cytokine (IL-10). Also, LDHMM caused some histopathological changes such as reactive gliosis and glia cell proliferation. LDHMM elevated the lead, cadmium and mercury concentrations in the brain. Severity of the distorted cortical parameters were ameliorated by CALE administration. The CALE induced significant protective effect on LDHMM-mediated neurotoxicity in a dose-dependent manner which may be a result of its antioxidant anti-inflammatory and metal chelation mechanisms.

## Highlights

• Low dose heavy metal mixture induced neurotoxicity marked by increased lipid peroxidation and pro-inflammatory cytokine (IL-6).
• Low dose heavy metal mixture induced neurotoxicity marked by lowered levels of the oxidative biomarkers (SOD, CAT and GSH) and anti-inflammatory cytokine (IL-10).
• Low dose heavy metal mixture also caused reactive gliosis and glia cell proliferation.
• LDHMM elevated the lead, cadmium and mercury concentrations in the brain.
• *Costus afer* aqueous leaf extract (CALE) treatment reversed all these effects.
• *Costus afer* aqueous leaf extract (CALE) may be neuroprotective.

## Graphical abstract

**Figure:**
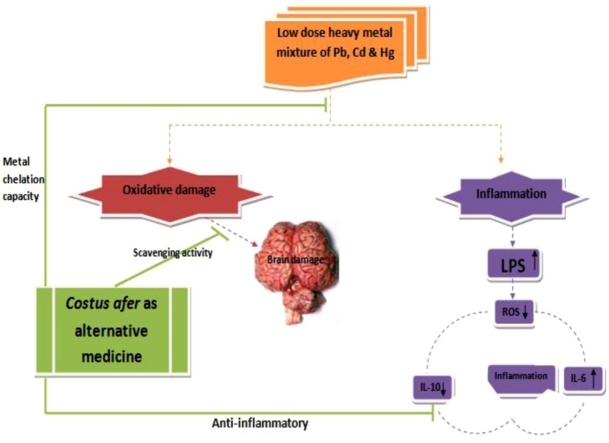

## 1 Introduction

Neurotoxicity refers to any adverse effect to the nervous system, caused by either a physical, chemical or biological agent thereby inhibiting the ability of an organism to live or adapt to its surrounding [1]. The deleterious effect arising from short-term exposure to neurotoxicants may be compensated by the brain, but a chronic exposure even at low concentration may cause delayed brain damage [2]. Pb, Hg, Cd and As are reported to show their neurotoxic effects [3,4] through common mechanisms, such as the production of reactive oxygen species (ROS) and interaction with micronutrients [3,5,6].

As a result of its capacity to scavenge ROS, an antioxidant- rich plant might be used to protect against its toxicity. Due to the polyphenolic content of many plants across the globe, herbal medicine has been proved to prevent degenerative diseases [7]. *Costus afer* (ker gawl) is an herbaceous plant belonging to the family Zingiberaceae. It has been revealed by many authors to have a wide range of therapeutic effects. The pharmacological activities associated with *Costus afer* include antioxidant property, hepatoprotectivity, nephroprotectivity, antidiabetic role [8,9] and antinociceptive role [10].

However, investigation into the possible protective effect of *Costus afer*aqueous leaf extract (CALE) on low dose heavy metal mixture (LDHMM) - mediated neurotoxicity has not been reported. Thus, the study evaluated the possible neuroprotective role of CALE in LDHMM (Pb, Cd and Hg) – mediated neuronal damage by estimating the levels of oxidative stress biomarkers, pro and anti-inflammatory cytokines and heavy metal concentrations in frontal cortex of male albino rats.

## 2 Methods

### 2.1 Collection and identification of *Costus afer*

*Costus afer* was harvested from the interior part of a farm land (away from vehicular traffic) behind the World Bank African Centre of Excellence for Oilfield Chemicals Research, University of Port Harcourt in Ikwerre Local Government Area of Rivers State, Nigeria. Identification and authentication of the plant material was done by Mr. A.O. Ozioko and given the voucher number [INTERCEDDO/033].

### 2.2 Preparation of the plant extract

Fresh leaves of *Costus afer* were collected, washed clean to eliminate any form of contamination and were pulverized. Then, 250 g of the pulverized leaves was weighed and macerated in 500 ml deionized water in a stoppered container and allowed to stand for 24 h with constant agitations at intervals following the previous work done by Ezejiofor and Orisakwe [9]. After vigorous shaking of the mixture, the pulverized leaves were pressed and the extract was separated. The filtered liquid was then stored in a refrigerator at 4 °C. The extract was redundant after the fourth day of treatment and fresh preparation was made. This process was continuous over the 90 days of treatment.

### 2.3 Animal husbandry

Forty-two young male Wistar rats, approximately 8 weeks old and weighing 100−200 g bought from the animal house of the Department of Experimental Pharmacology and Toxicology, University of Port Harcourt, Choba, Rives State, Nigeria were used for the study. The test animals were kept for fourteen days to adapt in polypropylene cages under 25 ± 2 °C, with relative humidity of 55–64 % and light and dark conditions (12/12 h) following the previous work of Ezejiofor and Orisakwe [9]. The protocol for the experiment was approved by the University of Port Harcourt Research Ethics Committee and the reference number UPH/CEREMAD/REC/04 was assigned. The animals were given standard feed and deionized water *ad libitum*.

### 2.4 Experimental design

Weight matched rats were divided into six groups of seven rats each. Group 1 served as control and received only deionized water, while group 2 was treated with heavy metal mixture (PbCl_2_, 20 mg/kg; CdCl_2_, 1.61 mg/kg; HgCl_2_, 0.40 mg/kg only) (Sigma Aldrich WGK Germany) according to the study by Institóris et al. [11]. Rats belonging to groups 3, 4 and 5 received the metal mixture and *Costus afer* extract at 750 mg/kg, 1500 mg/kg and 2250 mg/kg respectively according to Ezejiofor and Orisakwe [9]. Group 6 received the metal mixture and 0.80 mg/kg of an antioxidant metal ZnCl_2._

### 2.5 Sample collection

After the 90 days of treatment period, animals were sacrificed under ether anesthesia. The brain was weighed after excision. The relative brain weight was obtained by;

$$\text{R}\text{e}\text{l}\text{a}\text{t}\text{i}\text{v}\text{e}\;\text{w}\text{e}\text{i}\text{g}\text{h}\text{t}=\frac{\text{A}\text{b}\text{s}\text{o}\text{l}\text{u}\text{t}\text{e}\;\text{w}\text{e}\text{i}\text{g}\text{h}\text{t}}{\text{F}\text{i}\text{n}\text{a}\text{l}\;\text{b}\text{o}\text{d}\text{y}\;\text{w}\text{e}\text{i}\text{g}\text{h}\text{t}}\times \;100$$

The frontal cortex of the brain was processed for the assay of heavy metals and other biomarkers.

### 2.6 Metal analysis

Acid digestion of the brain was done by using 6 ml of Nitric acid and 2 ml of perchloric acid after isolating and weighing the organ. After acidification, the samples are placed for 30 min before heating at 105 °C until digestion is completed. The solution was then filtered with Whatmann filter paper to get a clear solution. The solution was later made up to 15 ml (final volume) with distilled water. Solar thermo elemental flame Atomic Absorption Spectrometer (Model SG 71906) was used to determine the levels of lead, cadmium and mercury in the frontal cortex of the brain.

### 2.7 Antioxidant analysis

#### 2.7.1 Catalase (CAT) activity

Catalase activity was estimated by slightly modifying the method by Clairborne [12]. This method is supported by the principle that catalase in the sample preparation split hydrogen peroxide which can be measured spectrophotometrically at 240 nm [12].

#### 2.7.2 Estimation of testicular reduced glutathione (GSH) level

The reduced glutathione level was estimated using the procedure explained by Sedlak and Lindsay [13].

#### 2.7.3 Estimation of Superoxide dismutase (SOD) activity

Superoxide dismutase activity was evaluated by using the method described by Misra and Fridovich [14]. This is principally based on the ability of SOD to inhibit the autoxidation of epinephrine at pH 10.2.

### 2.8 Lipid peroxidation marker (MDA) activity

The MDA level was assayed by using the procedure of Ohkawa and Ohishi [15]. Under acidic medium, MDA reacts with the chromogenic reagent, 2-thiobarbituric acid (TBA), to form a pink coloured complex at 532 nm absorbance.

### 2.9 Estimation of Inflammatory cytokines [Interlukin-6 (IL-6) and Interlukin-10 (IL-10)]

The activities of Il-6 and IL-10 were assayed using the enzyme linked immunosorbent assay kit (ELISA -Bioassay Technology Laboratory, 1713 Junjiang Inter. Bldg. 218 Ningguo Rd. Yangpu Dist. Shanghai, China) with the sensitivity of 0.052 ng/l and 1.51 pg/ml respectively. This process is principally based on immobilization.

### 2.10 Determination of hormone profile levels

The enzyme linked immunosorbent assay kit (IEMA/ELISA; EIA) - Accubind Elisa Microwells, Monobind Inc Lake Forest; CA 92630.USA was used in carrying out the activities of follicle stimulating hormone (FSH), luteinizing hormone (LH), testosterone (TET) and prolactin (PRL).

### 2.11 Statistical analysis

Data were analyzed by one-way analysis of variance (ANOVA) using SPSS package (23.0) version (SPSS Inc, USA); while multiple comparisons were done with Duncan’s multiple comparison method at 5 % significant level. All the results were expressed as Mean ± Standard deviation (S.D). A statistical tool, XLSTAT 2016 (Version 6 statistical package) was used to develop the regression models [16]. Pearson’s rank correlation of the inter-elemental relationship between toxic metals within brain of rats was also done.

## 3 Results

After 12 weeks of treatment, animals treated with only the metal mixture showed a marked elevation in brain weight (*p* < 0.05) in contrast to the control rats (treated with only deionized water). Rats that received a combination of heavy metal mixture and *Costus afer* at (750, 1500 and 2250 mg/kg respectively) and rats co-administered with zinc had significantly reduced brain weight compared to the heavy metal mixture-exposed rats (Table 1).

**Table 1:** Effects of *Costus afer* and zinc on the absolute and relative weight of brain on male albino rats treated with metal mixture.

Treatment	*Absolute (g)	Relative (%)
Deionized H_2_O (only)	1.54 ± 0.09^a^	0.74 ± 0.04^b^
Metal mixture (only)	1.84 ± 0.09^c^	0.71 ± 0.03^b^
Metal mixture +750 mg/kg	1.74 ± 0.09^bc^	0.72 ± 0.03^b^
Metal mixture +1500 mg/kg	1.72 ± 0.08^b^	0.65 ± 0.03^a^
Metal mixture +2250 mg/kg	1.62 ± 0.08^ab^	0.63 ± 0.03^a^
Metal mixture + Zinc	1.68 ± 0.08^b^	0.75 ± 0.03^b^

*Values are represented as (Mean ± SD, N=5). In the each column, values with different superscripts (a, b, c) are significantly different from each other (*p* <  0.05) and those with the same superscripts are not significantly different.

**Fig. 1:**
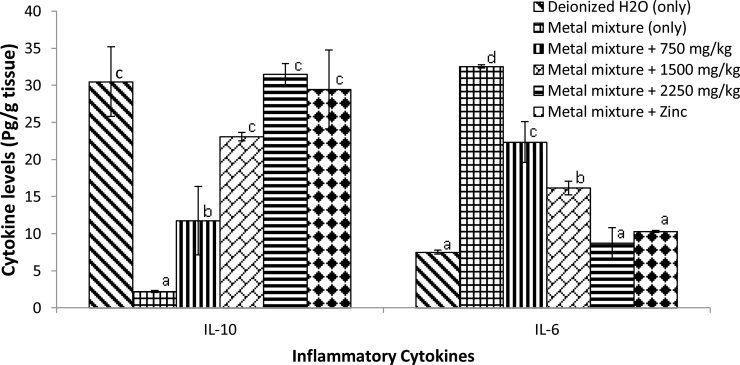
Effects of *Costus afer* and zinc on inflammatory cytokines (interlukin-10 (IL-10) and interlukin-6 (IL-6) on brain of male albino rats treated with metal mixture. Values with different superscripts (a, b, c) were significantly different from each other (*p* <  0.05) and those with the same superscripts were not significantly different.

Measuring the pro- and anti-inflammatory cytokine levels in the brain is essential in assessing the inflammatory status after metal mixture exposure. Co-administration of *Costus afer* and zinc significantly reduced and increased (*p* < 0.05) the levels of pro- and anti- inflammatory cytokines (IL-6 and IL-10) respectively when compared to those in heavy metal mixture-treated group (Fig. 1).

**Fig. 2:**
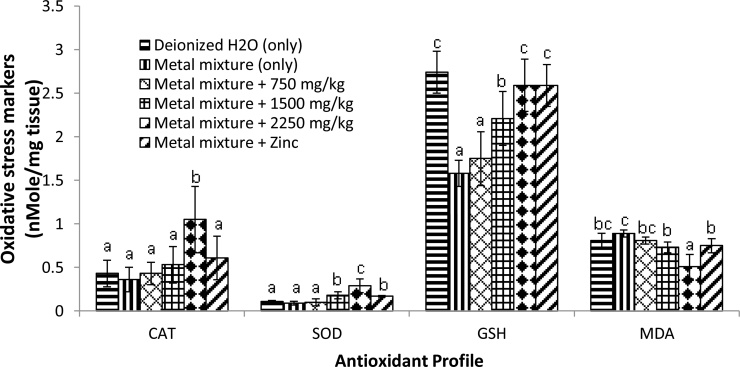
Effect of *Costus afer* and zinc on oxidative stress markers of the frontal cortex of the brain of male albino rats treated with heavy metal mixture. Values with different superscripts (a, b, c) were significantly different from each other (*p* <  0.05) and those with the same superscripts were not significantly different.

The MDA level was assessed in order to evaluate the oxidative status of the brain. Heavy metal mixture treatment at a dose of 20 mg/kg PbCl_2_, 1.61 mg/kg CdCl_2_ and 0.40 mg/kg HgCl_2_ body weight for 90 consecutive days induced changes in the oxidative state of the brain tissue. There was a marked increase in MDA level (*p* <  0.05) in the brain tissue of heavy metal mixture-intoxicated rats compared to those of rats in the control group (Fig. 2). Rats co-treated with *Costus afer* and zinc however, showed a reduction in MDA level compared to metal mixture-treated rats.

Concerning the effect on (GSH) and enzymatic (SOD and CAT) antioxidant system activities in brain, result in Fig. 2 shows that the exposure to low dose heavy metal mixture induced a marked decline (*p* < 0.05) in GSH content, SOD and CAT levels when compared with control rats. Rats co-treated with *Costus afer* and zinc showed elevation in GSH, SOD and CAT levels when compared with the heavy metal mixture-treated animals, representing the potent antioxidant capacities of *Costus afer* and zinc in the brain following heavy metal mixture-induced oxidative damage.

The concentrations of Pb, Cd and Hg in the frontal cortex of the brain were significantly increased (*p* < 0.05) in metal mixture-treated rats compared to the control rats (Table 2). However, the co-administration with *Costus afer* and zinc significantly reduced the levels of these heavy metals. Also, the result shows that the group treated with only heavy metal mixture showed the highest concentration of heavy metals (Pb = 78.906 ± 10.389, Cd = 1.543 ± 0.049 and Hg = 5.191 ± 0.287) compared to the control group (treated with deionized water only) having (Pb = 0.122 ± 0.012, Cd = <0.001 ± 0.000 and Hg = <0.001 ± 0.000).

**Table 2:** Concentration of heavy metals (mg/kg) in frontal cortex of the brain of male albino rats after exposure to heavy metal mixture with or without *Costus afer* and zinc.

Treatment	Cadmium (Cd)	Mercury (Hg)	Lead (Pb)
Deionized H_2_O (only)	<0.001 ± 0.000^a^	<0.001 ± 0.000^a^	0.122 ± 0.012^a^
Metal mixture (only)	1.543 ± 0.049^c^	5.191 ± 0.287^d^	78.906 ± 10.389^d^
Metal mixture +750 mg/kg CA	0.274 ± 0.200^b^	3.873 ± 0.389^c^	39.278 ± 5.966^c^
Metal mixture +1500 mg/kg CA	0.055 ± 0.033^a^	1.534 ± 0.385^b^	12.063 ± 1.308^b^
Metal mixture +2250 mg/kg CA	0.002 ± 0.001^a^	0.007 ± 0.001^a^	3.507 ± 1.560^ab^
Metal mixture + Zinc	0.002 ± 0.001^a^	0.018 ± 0.021^a^	2.538 ± 1.289^a^

*Values are expressed as (Mean ± SD). Values in the same column with different superscripts are significantly different from each other (*p* < 0.05) and those with the same superscripts in the same column are not significantly different; where CA = *Costus afer.*

**Fig. 3:**
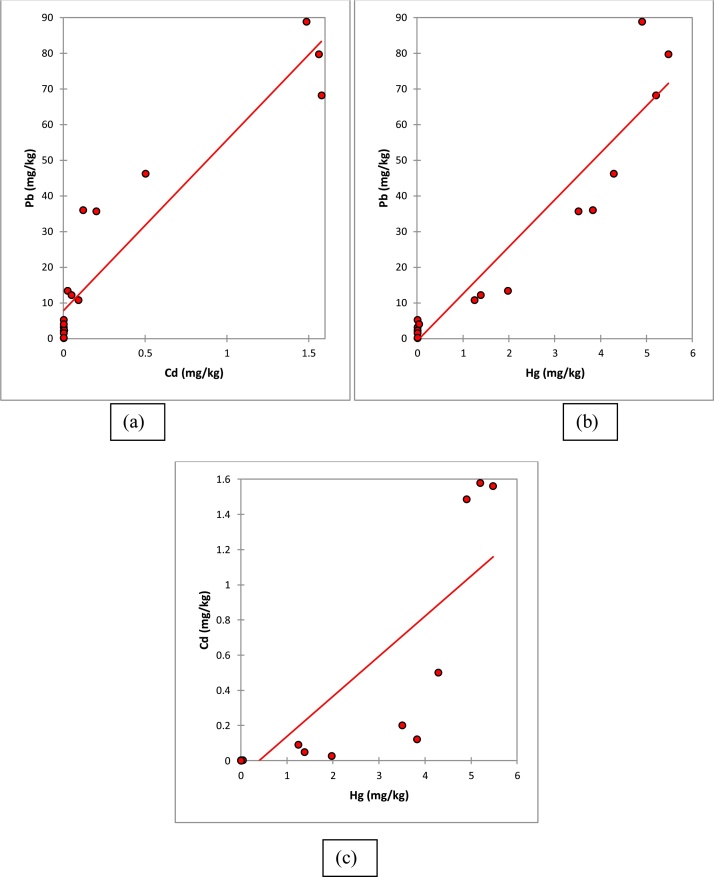
Inter-elemental correlation among toxic metals within brain of rats showed strong positive correlation (r > 0.80) between metals such as (a) Cd and Pb (b) Hg and Pb (c) Hg and Cd during the study. All correlations were significant at *p* < 0.01.

Pearson’s rank correlation showed the inter-elemental relationship between toxic metals within brain of rats with strong positive correlation (r > 0.80) between (a) Cd and Pb (b) Hg and Pb (c) Hg and Cd. All correlations were significant at *p* < 0.01 (Fig. 3).

The four independent parameters (Pb, Cd, Hg and *Costus afer*) formed the input data and were allowed to pass through multiple linear regressions for purpose of model calibration. The output indicates zero coefficients for x_2_ and x_3_. Thus, subsequent model calibration excluded the three constant parameters which formed the input data. In effect, y becomes a function of *Costus afer* variable for a given set of Pb, Cd, and Hg variables. Subsequently, concentration of catalase (y) against *Costus afer* (x) was subjected into trial models of linear, quadratic and exponential options (Table 3). The process is repeated for other parameters and the best with respect to goodness of fit (R^2^), mean square error (MSE) and root mean square error (RMSE) values were selected and summarized as shown in Table 4 for all experiments carried out on the brain.

**Table 3:** Summary of regression models for catalase (CAT) in brain.

Model Type	Equation	R^2^	MSE	RMSE
Exponential	*y* = 10.324e^0.000x^	0.887	0.012	0.110
Linear	*y* = 0.000*x* + 0.267	0.801	0.029	0.171
*Quadratic	*y* = 2E-07*x*^2^-0.000*x*+0.379	0.974	0.008	0.087

*The best model with respect to R^2^, MSE and RMSE values.

A repetition of regression models was carried out on other parameters and the best with respect to R^2^, MSE and RMSE values was selected and summarized as shown in Table 4.

**Table 4:** Model equations for antioxidant biomarkers and inflammatory cytokines in the brain.

Parameters	Model Type	Model Equations	R^2^	MSE	RMSE
SOD	Quadratic	*y* = 4E-08*x*^2^-9E-06*x*+0.088	0.996	0.000	0.009
GSH	Quadratic	*y* = 9E-08*x*^2^+0.000*x*+1.561	0.989	0.007	0.083
MDA	Quadratic	*y*= -6E-08*x*^2^-2E-05*x*+0.883	0.987	0.001	0.031
Inflammatory cytokines					
IL-6	Quadratic	*y* = 1E-06*x*^2^-0.013*x*+32.29	0.995	1.352	1.163
IL-10	Linear	*y* = 0.013*x*+2.25	0.997	0.729	0.854

Where *y* = concentration of the parameter analyzed, *x* = *Costus afer* dose, MSE = Mean Squared Error, RMSE = Root Mean Squared Error.

**Fig. 4:**
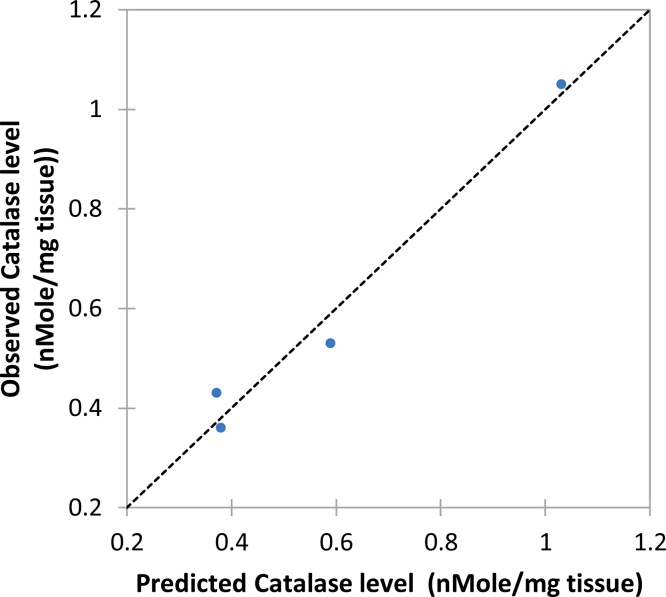
Predicted brain catalase level (nMole/mg tissue) against observed brain catalase level; where *y* = Catalase levels; *x* = *Costus afer* concentrations served as input values.

To verify the calibrated models, it was imperative to compare the observed against predicted testicular catalase level (Fig. 4) with corresponding goodness of fit, R^2^ = 0.974. Similarly, the verification was repeated for other test parameters in the brain. Hence, each model is essential to forecast the applicable dependent variable for a given independent variable (*Costus afer*) at constant Pb, Cd and Hg concentrations as used in this study.

The histologic observations in the frontal cortex of the brain showed no obvious change in rats treated with only deionized water and reactive gliosis was observed in rats treated with only the heavy metal mixture. Mild signs of reactive gliosis was observed in rats co-treated with 750 mg/kg of *Costus afer*, while mild glial cell proliferation was obvious in rats co-treated with 1500 mg/kg of *Costus afer*. Rats co-treated with 2250 mg/kg of *Costus afer* and 0.80 mg/kg of zinc showed no obvious histologic change (Table 5).

**Table 5:** Histologic observations in the frontal cortex of the brain of male rats exposed heavy metal mixture with or without *Costus afer* and zinc.

Treatment groups	Histopathologic Observations
Deionized water (only)	No obvious histologic change
Heavy metal mixture (only)	Reactive gliosis observed
Heavy metal mixture +750 mg/kg CA	Mild signs of reactive gliosis observed
Heavy metal mixture +1500 mg/kg CA	Mild glial cell proliferation observed
Heavy metal mixture +2250 mg/kg CA	No obvious histologic change
Heavy metal mixture +0.80 mg/kg ZnCl_2_	No obvious histologic change

*CA = *Costus afer* and ZnCl_2_ = Zinc chloride.

## 4 Discussion

Lead (Pb), cadmium (Cd), and mercury (Hg) are some of the most toxic metals humans are exposed to which target essential organs including brain [17] are of significant public health concerns [18]. In this study rats exposed to low dose heavy metal mixture of Pb, Cd and Hg showed an increase in the absolute weight of the brain compared to the control group. Since a significant increase or decrease in either absolute or relative weight of an organ after administering a chemical signifies the noxious effect of that chemical [19,20], this study confirms that the brain could be a target organ over a long-term exposure to low dose mixture of Pb, Cd and Hg. Treatment with *Costus afer* reversed the effect of the low dose heavy metal mixture on the absolute and relative weights of the brain in a dose dependent manner. The concomitant treatment with zinc also caused a significant decrease (*p* <  0.05) in the absolute brain weight compared to the toxic control group which is akin to the effect of *Costus afer* extract. *Costus afer* like zinc may be protective against low dose heavy metal mixture induced neurotoxicity in rat.

Similarly, long-term exposure to low dose heavy metal mixture of Pb, Cd and Hg caused an imbalance in immune regulation leading to a marked increase (*p* <  0.05) in the pro-inflammatory cytokine [interlukin-6 (IL-6)] and a significant reduction (*p* <  0.05) in anti-inflammatory cytokine [interlukin-10 (IL-10)] compared to the control group. On the other hand, these observations were reversed in a dose dependent manner by concomitant treatment with *Costus afer*. Interestingly both *Costus afer* and zinc an essential trace element produced the same anti-inflammatory effects on the brain. The up-regulation of anti-inflammatory cytokine [interlukin-10 (IL-10)] and down regulation of pro-inflammatory cytokine [interlukin-6 (IL-6)] by *Costus afer* suggests beneficial role of this plant extract. Cytokines attract immune cells to the site of injury or infection. Due to the ability of oxidative stress to induce several health problems, it activates persistent neuroinflammation, distinguished by the development of pro-inflammatory mediators locally produced by host cells, thus engaging the intrinsic immune system [21]. When the generation of free radicals surpasses the counteracting effects of endogenous antioxidants, ROS becomes extremely noxious to the central nervous system. Neuroinflammation supports oxidative stress and add to loss of cells and permanent neuronal dysfunction [22], even though neuroinflammation can add to the outcome of unremitting oxidative stress. The pro-inflammatory cytokines including IL-6 are potentially essential or deleterious, depending on their concentration, site and duration of action [23] and they draw out immune responses in the central nervous system (CNS) during inflammation. Probably, elevated pro-inflammatory cytokine signaling may encourage ROS production resulting to oxidative damage, and this may be one mechanism that relates inflammation to neuropsychiatric diseases [21].

In this study, there were severe biochemical and neurochemical changes in the brain resulting from exposure to low dose heavy metal mixture of Pb, Cd and Hg. Rats treated with only low dose heavy metal mixture (20 mg/kg PbCl_2_, 1.61 mg/kg CdCl_2_ and 0.40 mg/kg HgCl_2_ mg/kg body weight) for 90 days showed oxidative stress marked by increase in MDA content and decrease in the activities of CAT, SOD and GSH. These observations are in line with the works of Radwan et al. [24] who reported that exposure to a heavy metal caused a marked elevation in MDA level and decrease in SOD, CAT and GSH contents in the brain. Our findings are also consistent with the previous works of Karaca and Eraslan [25] and Abdel Moneim [26] which reported that exposure to heavy metals could lead to reactive oxygen species (ROS) generation, causing an elevation in MDA level, sulfhydryls depletion, changes of antioxidant cellular defenses and DNA damage. The elevation in the lipid peroxidation marker could be from overproduction of the superoxide anions which stifle the antioxidant enzymatic system [27]. The decreased activities of SOD and CAT levels may be because of binding of the heavy metals with the sulfhydryl group of these enzymes and the substitution of endogenous redox metals which changes these enzyme configurations leading to their inhibition [28].The depletion in the GSH content may be attributed to the use of GSH in scavenging the generated free radicals. The combined effect of increased MDA and decreased SOD, CAT and GSH in our present study could lead to neurodegeneration as a result of heavy metal mixture exposure. Nevertheless, the results suggested that treatment with varying doses of *Costus afer* (750, 1500 and 2250 mg/kg body weight) significantly alleviated the brain oxidative status induced by heavy metal mixture exposure. This could be ascribed to its antioxidant properties.

The significant reduction in the brain levels of lead (Pb), cadmium (Cd) and mercury (Hg) following the administration of *Costus afer* is noteworthy. Although the exact mechanism is obscure but chelation of these metals by an active ingredient of this natural antidote is clear. Recent developments in oxidative stress and understanding of its management require a dual function of the antioxidants namely metal chelation and free radical scavenging [[29], [30], [31]]. The above proven antioxidant mechanism of *Costus afer* suggest the presence of metal ion chelating activity of an antioxidant moiety which prevents oxyradical generation and consequent oxidative damage [32]. Further studies will be needed to understand the exact mechanism of action and the levels of these metals in urine and fecal matter to ascertain their routes of elimination.

Pearson’s rank correlations of toxic metals (Pb, Cd and Hg) presents very positive significant correlations (r > 0.80) between Cd-Pb (r = 0.940, p < 0.05, n = 18), Hg-Pb (r = 0.953, p < 0.05, n = 18), Hg-Cd (r = 0.840, p < 0.05, n = 18) in the brain. The strong relationships existing between these toxic metals in brain tissue of rats exposed to low dose heavy metal mixture may implicate a close physiological correlation. The strongest correlation was observed in the association between brain Hg and brain Pb. These strong correlations between the toxic metals could also be attributed to the similar oxidative states of the toxic metals, suggestive of similar mechanisms.

Reactive gliosis (a reaction of the central nervous system to injury of the brain or spinal cord) is significant in rats treated with low dose heavy metal mixture of Pb, Cd and Hg compared to rats treated with only deionized water which showed no obvious histologic change after 90 days of treatment. A study by Adedayo et al. [33] confirmed that heavy metals such as lead (Pb) induce inflammation and neurodegenerative changes in the rat medial prefrontal cortex after exposure for 21 days. Significantly, the overexpression of IL-6 in the development of the brain could adversely affect the growth and differentiation of neurons via reactive gliosis (increased size and the number of astrocytes and ramified microglia), and may have an activating effect on N-methyl-d-aspartate receptors (NMDA) receptors in neurons, causing excessive activation of nerve cells, which in turn would lead to their death by necrosis [34]. Rats treated with a combination of heavy metal mixture and *Costus afer* at 750 mg/kg showed signs of reactive gliosis. Rats treated with a combination of heavy metal mixture and *Costus afer* at 1500 mg/kg showed mild glial cell proliferation, while the histologic slides from the brain of rats co-treated with metal mixture plus *Costus afer* (2250 mg/kg) and zinc revealed no obvious histologic change in the neural cells, molecular zone, granular zone and white matter. This result indicates that exposure to metal mixture even at low dose could cause some pathological changes in the central nervous system and these changes could be attenuated with the aqueous leaf extract of *Costus afer* and zinc. Costus afer contains 4.09–4.75 mg/100 g zinc [35] which may contribute at least in part to the beneficial effect seen in this study.

The predictive models are useful in forecasting the residual values of antioxidant profile and inflammatory cytokines in the brain of male albino rats at any treatment dose with *Costus afer* to a high precision at constant Pb, Cd and Hg concentrations as used in this study. The models will help to save time and resources in the treatment of LDHMM – induced toxicity using *Costus afer*. The linear and exponential models had R^2^, MSE and RMSE of (0.801, 0.029 and 0.171) and (0.887, 0.012 and 0.110) respectively. The quadratic model was the best model with respect to goodness of fit; R^2^, mean square error (MSE), and root mean square error (RMSE) values (i.e. 0.974, 0.008 and 0.087). A repetition of the regression models was done for other parameters and based on R^2^, MSE and RMSE, the best models were selected.

To verify the calibrated models, it was necessary to compare observed against predicted brain catalase level with corresponding goodness of fit, R^2^ = 0.974. The modeled brain catalase values for *Costus afer* co-treated groups of male albino rats at 0 mg/kg, 750 mg/kg, 1500 mg/kg and 2250 mg/kg were 0.380, 0.372, 0.589 and 1.031, while the observed values were 0.360, 0.430, 0.530 and 1.050 respectively. Similarly, the verification was done for other parameters in the brain. Hence, each model can be used to predict the applicable dependent variable for a given independent variable (*Costus afer*) at constant Pb, Cd and Hg concentrations as used in this study.

## 5 Conclusion

Low dose heavy metal mixture caused oxidative damage in the brain which is evidenced by a change in antioxidant markers (SOD, CAT and GSH) and an increase in the lipid peroxidation marker (MDA). There was up regulation of pro-inflammatory and down regulation of anti-inflammatory cytokines, elevated levels of lead, cadmium and mercury in the brain and histopathological changes including reactive gliosis following exposure to low dose heavy metal mixture. The *Costus afer* aqueous leaves extract (CALE) significantly reversed all these effects in dose dependent fashion. *Costus afer* and zinc may be beneficial in low dose heavy metal mixture-induced neurodegeneration in rats by its antioxidant and anti-inflammatory mechanisms.

## CRediT authorship contribution statement

**Brilliance O. Anyanwu:** Data curation, Formal analysis, Funding acquisition, Investigation, Resources, Writing - original draft, Writing - review & editing. **Chinna N. Orish:** Methodology, Project administration, Validation, Visualization. **Anthonet N. Ezejiofor:** Methodology, Project administration, Supervision, Validation, Visualization. **Ify L. Nwaogazie:** Data curation, Formal analysis, Software. **Orish E. Orisakwe:** Conceptualization, Formal analysis, Methodology, Project administration, Supervision, Validation, Visualization, Writing - original draft, Writing - review & editing.

## Declaration of Competing Interest

The authors report no declarations of interest.
